# Efficacy and safety of mini percutaneous nephrolithotomy in obese patients

**DOI:** 10.1186/s40064-016-2830-0

**Published:** 2016-07-22

**Authors:** Fatih Akbulut, Onur Kucuktopcu, Emre Kandemir, Burak Ucpinar, Faruk Ozgor, Abdulmuttalip Simsek, Burak Arslan, Akif Erbin, Fatih Yanaral, Murat Binbay, Gokhan Gurbuz

**Affiliations:** Department of Urology, Haseki Training and Research Hospital, Istanbul, Turkey

**Keywords:** Mini percutaneous nephrolithotomy, Obesity, Surgical outcomes, Complications, Safety

## Abstract

**Purpose:**

We aimed to evaluate the effect of obesity on surgical outcomes of mini percutaneous nephrolithotomy (Mini-PNL).

**Methods:**

Hundred and eighty two Mini-PNL procedures were performed between May 2013 and January 2015 and their results were evaluated retrospectively. Patients were classified as non-obese (BMI, 18.5–30 kg/m^2^) and obese (≥30 kg/m^2^) groups. Obese and non-obese patients were compared according to pre-operative demographic values, intra-operative surgery techniques and post-operative results.

**Results:**

BMI values of 133 patients were lower than 30 kg/m^2^ while 49 patient’s BMI values were higher than 30 kg/m^2^. There were no significant difference between operation time, fluoroscopy time, number of access and access sites when two groups were compared. No significant difference was found in total length of hospital stay, hemoglobin drop, and complication rates. Stone-free rates were 70.7 % in the non-obese and 71.4 % in the obese group (p = 0.9).

**Conclusions:**

Mini-PNL procedure is a safe and effective treatment modality, which should be strongly considered for obese patients with appropriate sized stones.

## Background

Obesity is a serious health problem that arises due to world’s changing social and economic status and physical inactivity (Shikora 2005). Obese patients have increased risk of having diseases like; diabetes mellitus, hypertension, cardiovascular diseases, obstructive sleep apnea syndrome, and urinary stone disease (Taylor et al. 2005; Stamatelou et al. 2003; Curhan et al. 1998). Increased insulin resistance, hyperinsulinemia, hypocitraturia, hyperoxaluria and hypercalcemia can be listed as possible causes of increased incidence of stone disease among obese patients (Goldfarb 2003; Yang and Bellman 2004; Curhan 2007). Thus, treatment of stone disease in obese patients may be more important health issue in the near future.

Shock wave lithotripsy (SWL) treatment in obese patients appears to be less effective due to increased skin-to-stone distance and difficulties in performing the procedure (Thomas and Cass 1993; Al-Hayek et al. 2013). Performing flexible URS (F-URS) in obese patients with high stone burden can result in increased amount of residual stone fragments (Fabrizio et al. 1998). Residual stone fragments may be an obstacle to optimal treatment of obese patients who have increased risk of having stone disease.

Percutaneous nephrolithotomy is a commonly performed procedure in obese patients, even though it has certain problems like; bleeding, pain, and difficulties in positioning of the patient. Although several studies indicate that PNL is a reasonable option in obese patients (Faerber and Goh 1997; Carson et al. 1988; El-Assmy et al. 2007; Koo et al. 2004), there are also studies, which show increased risk of bleeding, technical difficulties in patient positioning, and difficulties in surgery techniques (Bagrodia et al. 2008; Desai et al. 2004; Sergeyev et al. 2007; Olbert et al. 2007).

Bleeding which requires transfusion due to conventional PNL procedure is one of the serious problems that urologists encounter. Transfusion rates can be as high as 21 % in morbid obese patients (Pearle et al. 1998). According to one study, tract dilatation technique and diameter of the tract are listed as factors that affect the risk of bleeding (Kukreja et al. 2004). After the invention of mini-PNL, the size of working sheaths decreased. This process resulted in decreased hemorrhage-related complications and hospitalization times with similar stone-free rates. Due to these reasons; mini-PNL procedure, which is performed through a narrower and shorter tract, is preferred in appropriate cases (Helal et al. 1997). Today, with the development of different size instruments there is a confusion of terminology used to describe the procedures. However conventional PNL is done through the tract size >24 Fr. Thus, mini-PNL terminology is used for the PNL procedures done through 22–14 Fr-tract size (Turk et al. 2015). On the other hand, length of mini PNL instruments may not be long as to reach to desired calyx or to allow manipulations in the kidney, in obese patients. To the best of our knowledge, no previous study specifically investigated the safety or efficacy of mini-PNL in obese subjects. In this study, we aimed to compare the safety and efficacy of mini-PNL procedure between non-obese and obese patients.

## Methods

One hundred and eighty-two mini-PNL procedures were performed between May 2013 and January 2015 and their results were evaluated retrospectively. The patients were divided into two groups according to their BMI values. Patients with a BMI value lower than 30 kg/m^2^ were defined as the non-obese group, and those with a BMI of ≥30 kg/m^2^ were defined as the obese group. Patients were evaluated pre-operatively with urinalysis, urine culture, blood counts, intravenous pyelography (IVP) and computed tomography (CT). Appropriate antibiotics were prescribed to patients with positive urine cultures until a sterile urine culture was obtained. Patients younger than 18 years of age and patients with coagulopathy were excluded from our study. The groups were compared according to their pre-operative demographic values and intraoperative and post-operative results. Patients with radio-opaque stones and non-radio-opaque stones were evaluated with kidney-ureter-bladder (KUB) X-ray and/or ultrasonography on post-operative day 2, respectively and all patients were evaluated regarding success rate with kidney-ureter-bladder (KUB) X-ray and ultrasonography or CT on the first post-operative month. Patients with complete stone clearance and patients with residual fragments under 4 mm were accepted as stone free.

### Mini-PNL technique

Under general anesthesia in the lithotomy position, a 5-Fr ureteral catheter was inserted and fixed to the 16-Fr bladder catheter. The calyceal system was visualized using contrast media in the prone position. Access was performed using an 18-G percutaneous access needle under the fluoroscopy. A 0.035-inch hydrophilic guidewire was inserted, and a second guidewire was inserted with a dual lumen catheter. Dilatation was performed using Amplatz dilatators up to 18Fr. After placement Amplatz sheath to the collecting system over the dilator, nephroscopy was done with 12 or 17 Fr nephroscope (Karl Storz, Tuttlingen, Germany) and stone fragmentation was performed using a laser, pneumatic or ultrasonic lithotripter. Stone extraction was performed using a 5-Fr stone extraction device. The surgery was finalized after insertion of a 14-Fr nephrostomy tube under fluoroscopy.

### Statistical analysis

The variables were expressed as numbers, percentage, mean values, and standard deviations. Comparisons were performed by Chi square test, Student *t* test, and Mann–Whitney U test. The significance of the relationships between the variables was analyzed using Pearson correlation analysis.

## Results

Pre-operative demographic values are shown in Table 1. The non-obese group consisted of 133 and the obese group consisted of 49 patients. The subjects in the non-obese group were younger (p < 0.001). Average stone size was similar between the groups (23.9 ± 9 mm and 26.2 ± 8.6 in the non-obese and obese groups, respectively, p = 0.13). There was no significant difference between two groups in creatinine values, stone locations, operation side, hydronephrosis grade, opacity of stones, and history of operations and SWL.

**Table 1:** Comparison of pre-operative values

	Body Mass Index				p values
	<30		≥30		
		%		%	
Number of patients	133		49		
Sex					0.004
Male	91	68.4	22	44.9	
Female	42	31.6	27	55.1	
Mean age (years)	41.8 ± 12.6 (18–78)		51.7 ± 12.5 (19–76)		<0.001
Mean Body Mass Index (kg/m^2^)	25.5 ± 4.3 (17.8–29.8)		34.3 ± 4.3 (30.0–46.4)		<0.001
Mean stone size (mm)	23.9 ± 9.0 (5–60)		26.2 ± 8.6 (15–60)		0.134
Localization of stones					
Multiple calyces	67	50.4	27	55.1	
Pelvis	25	18.8	8	16.3	
Lower calyces	29	21.8	12	24.5	
Middle calyces	7	5.3	0	0	
Upper calyces	5	3.8	2	4.1	
Side of the operated kidney					
Left	72	54.1	30	61.2	
Right	61	45.9	19	38.8	
Grade of hydronephrosis					0.802
0	4	3	1	2.1	
1	76	57.6	31	64.6	
2	44	33.3	15	31.3	
3	7	5.3	1	2.1	
4	1	0.8	0	0	
Opacity					0.050
Non-opaque	3	2.3	4	8.2	
Opaque	129	97	43	87.8	
Semi-opaque	1	0.8	2	4.1	
Previous ESWL history	29	23.6	17	37.0	0.082
Previous PNL history	46	37.1	20	46.5	0.277
Previous open surgery history	26	21	8	17.8	0.648

*BMI* Body Mass Index

Intra-operative findings of groups were summarized in Table 2. There was no statistical difference between two groups in operation time, fluoroscopy time, number of accesses, and access sites. Mean operation time was 111 min in the non-obese group and 109 min in the obese group (p = 0.805). Stone-free rates were 70.7 % in the non-obese group and 71.4 % in the obese group (p = 0.9). There was no significant difference in length of hospital stay, hemoglobin drop, or post-operative complications (Table 3). Body mass index was not correlated with any of the study variables (Table 4).

**Table 2:** Comparison of intra-operative values

	BMI				p values
	<30		≥30		
		%		%	
Number of patients	133		49		
Mean operation time (minutes)	111.4 ± 42.5 (45–250)		109.6 ± 41.6 (40–240)		0.805
Mean fluoroscopy time (minutes)	5.4 ± 3.4 (0.7–18.8)		5.8 ± 4.7 (0.8–20)		0.471
Number of access					0.415
1	123	92.5	43	87.8	
2	9	6.8	6	12.2	
3	1	0.8	0	0	
Access site					0.486
Multiple	7	5.4	4	8.2	
Lower calyces	105	78.9	41	83.7	
Middle calyces	17	12.8	3	6.1	
Upper calyces	4	0.8	1	2.0	

*BMI* Body Mass Index

**Table 3:** Comparison of post-operative values

	BMI				p values
	<30		≥30		
		%		%	
Number of patients	133		49		
Mean hospitalization duration (hours)	65.7 ± 28.8 (24–192)		61.3 ± 47.1 (24–192)		0.454
Mean hemoglobin drop (g/dl)	0.78 ± 1.12 (0–4.8)		1.08 ± 1.2 (0–4.2)		0.139
Post-operative complications					
Fever	1	0.8	1	2.0	0.459
Transfusion	4	3.0	1	2.0	0.723
Angioembolisation	1	0.8	0	0	0.543
Pulmonary complications	0		0		
Post-operative DJ insertion	24	18	6	12.2	0.350
Results					0.921
Number of patients with residual stones	39	29.3	14	28.6	
Number patients with stone-free status	94	70.7	35	71.4	

*BMI* Body Mass Index

**Table 4:** Summary of univariate and multivariate anaylsis, which shows the effect of obesity on operation outcomes

	BMI	p values
Mean operation time (min)	0.805	0.486
Mean fluoroscopy time (min)	0.471	0.635
Mean hospitalization duration (h)	0.454	0.782
Mean hemoglobin drop (g/dl)	0.139	0.043
Post-operative complications		
Fever	0.462	0.638
Transfusion	0.725	0.955
Angioembolisation	0.545	0.473
Post-operative DJ insertion	0.352	0.917
Result	0.922	0.54

*BMI* Body Mass Index

## Discussion

Obesity appears to be a serious health problem in multiple ways and its incidence is significantly rising in the last 50 years. Risk of renal stone disease is high in obese patients due to hyperinsulinemia related acidic urine and disordered ammonium metabolism and disorders of renal tubules (Asplin 2009; Sakhaee and Maalouf 2008).

Shock wave lithotripsy, F-URS, open surgery, PNL and mini-PNL are treatment options for renal stone disease. SWL is not a reasonable option in obese patients due to increased skin-to-stone distance, inability to maintain a clear vision during fluoroscopy, and technical difficulties in positioning of the patient (Robert et al. 1999; Munoz et al. 2003; Alkan et al. 2015). Open surgery is not routinely preferred due to increased risk of surgery site infections, post-operative pain, thromboembolism, and respiratory problems; prolonged healing time; and longer incision and scar formation (Choban and Flancbaum 1997).

Recently, F-URS is an outstanding treatment method due to its natural orifice transluminal endoscopic surgery (NOTES) feature. F-URS has been shown to be effective in treatment of kidney stones, which are less than 2 cm but operation time is longer and multiple operations may be needed needed for complete stone clearance (Nguyen and Belis 1998; Sergeyev et al. 2007). These problems limit the usage of F-URS in obese patients because prolonged operation times and multiple procedures can increase the risk of morbidity and mortality in these subjects (Oberg and Poulsen 1996; Adams and Murphy 2000; Bond 1993; Postlethwait and Johnson 1972; Perberton and Manax 1971).

Notably, PNL is a preferable method in the treatment of renal stone disease in obese patients compared with other treatment modalities. But it is clear that there are certain difficulties of the PNL procedure, especially in obese patients. Difficulty of positioning of the patient in the prone position, increased risk of pressure ulcer formation, and the potential need of increased rates of transfusion can be listed as some of these difficulties.

Mini-PNL, which was first defined for pediatric population by Helal et al. (1997), is a new treatment option for obese patients, with its lower risk of parenchymal injury and smaller tract size. There are no prior studies in literature about safety and efficacy of mini-PNL in obese patients. In a CROES study published by Fuller et al. (2012), conventional PNL was performed in 5803 patients who were stratified into 4 different groups according to their BMIs. Stone-free rates were 78.9 % in the obese group and 65.6 % in the morbid obese group. In a different study about efficacy of F-URS in obese patients published by Sari et al. (2013), stone-free rates were 73.6 % in obese and 61.5 % in morbid obese group. In a similar study about efficacy of F-URS in obese patients with stones greater than 2 cm published by Alkan et al. (2015), stone-free rates were 75.6 % in obese and 66.6 % in morbid obese patients. In a similar study by Carson et al. (1988), 44 obese and 226 non-obese patients were compared and no statistical difference was identified in operation times, the need for multiple procedures to achieve complete clearance, total length of hospital stay, complication rates, or stone-free rates. In our study, stone-free rates were 70.7 % in the non-obese group and 71.4 % in the obese group.

In a CROES study about conventional PNL by Fuller et al. (2012), complication rates in obese and morbid obese patients were 18.5 and 22.1 %, respectively. In a study by Simsek et al. (2014), conventional PNL was performed in 2012 patients and these patients were stratified into 4 groups according to their BMIs. 370 patients had a BMI value greater than 30 kg/m^2^. 20 patients (5.4 %) developed pelvicalyceal perforation, 5 patients had pulmonary problems (1.3 %), 2 patients developed cardiac problems (0.5 %), and 1 patient experienced hemorrhage, which required nephrectomy (0.2 %). In our study, the complication rate was 12.2 % in the obese group and pelvicalyceal perforation was seen in 4 patients (2.1 %). There were no pulmonary complications or hemorrhage requiring nephrectomy.

Bleeding is a frequent complication of PNL and management of bleeding is crucial. According to studies that have included large patient series, bleeding which requires transfusion can be seen in 5–18 % of cases and bleeding which requires embolization can be seen in 0.3–1 % (Michel et al. 2007; Skolarikos and de la Rosette 2008; Kessaris et al. 1995; Turna et al. 2007; El-Nahas et al. 2007). Imaging modality during percutaneous access (ultrasonography vs. fluoroscopy), tract dilatation technique, tract size, and tract length are all factors, which can affect bleeding rates in PNL (Pearle et al. 1998). In the light of this information, mini-PNL seems to be a preferable option due to its lesser parenchymal injury formation. In a study by Ates et al. (2011), evaluated the safety of PNL on 194 patients and bleeding rates in obese and non-obese patients were 12.3 and 12.9 %, respectively. In the CROES study, conventional PNL was performed in patients and bleeding rates in obese and morbid obese patients were 4.5 and 5.2 %, respectively (Fuller et al. 2012). In our study, mean hemoglobin drop in the non-obese and obese groups were 0.78 and 1.08 mg/dl, respectively. The rates of transfusion requirements were similarly low in the two groups. Transfusion rates and bleeding rates in our study were lower when compared to conventional PNL data in the literature.

Our study has several limitations, including the retrospective design of the study, presence of multiple surgeons instead of a single surgeon, absence of stone analysis results and evaluation of stone-free status. It is well known that KUB and ultrasonography are not as sensitive as computed tomography in the detection of residual stone fragments, especially in obese subjects. Although CT examination would provide a more accurate success rate in obese subjects, the stone-free status was assessed with KUB and ultrasonography due to concerns about the radiation exposure.

## Conclusions

Our study has demonstrated that efficacy and safety of the mini-PNL procedure were similar in obese and non-obese groups. Mini-PNL procedure seems to be a safe and efficacious option in obese patients with appropriate sized stones.
